# Doença de Kawasaki: Preditores de Resistência à Imunoglobulina Intravenosa e Complicações Cardíacas

**DOI:** 10.36660/abc.20190758

**Published:** 2021-03-03

**Authors:** Diogo Faim, Cláudio Henriques, Ana Brett, Andreia Francisco, Fernanda Rodrigues, António Pires

**Affiliations:** 1 Centro Hospitalar e Universitário de Coimbra EPE Coimbra Portugal Centro Hospitalar e Universitário de Coimbra EPE - Cardiologia Pediátrica, Coimbra - Portugal; 2 Centro Hospitalar e Universitário de Coimbra EPE Coimbra Portugal Centro Hospitalar e Universitário de Coimbra EPE - Urgência e Unidade de Infeciologia, Coimbra - Portugal

**Keywords:** Doença de Kawasaki/complicações, Síndrome de Linfonodos Mucocutâneos/complicações, Resistência à Medicamentos, Doenças da Artéria Coronariana, Imunoglobulina, Criança

## Abstract

**Fundamento:**

A doença de Kawasaki (DK) é a principal causa de cardiopatia adquirida em idade pediátrica nos países desenvolvidos.

**Objetivos:**

Identificar fatores preditores de resistência à imunoglobulina intravenosa (IGIV), calcular a eficácia dos modelos preditores japoneses e caracterizar as complicações cardíacas.

**Métodos:**

Análise retrospectiva dos casos de DK entre janeiro de 2006 e julho de 2018 em um hospital pediátrico português. Foram construídas curvas ROC para encontrar fatores preditores de resistência e utilizada regressão logística multivariada para elaborar o modelo preditor. O nível de significância utilizado foi de 5%.

**Resultados:**

Foram incluídos 48 pacientes com mediana de idade de 36 meses. Verificou-se resistência à IGIV em 21%. Ocorreram alterações ecocardiográficas em 46%, com envolvimento coronário em 25%. Como variáveis preditoras de resistência, a proteína C-reativa (PC-R) apresentou uma AUC ROC = 0,789, ponto de corte = 15,1 mg/dL, sensibilidade (S) = 77,8% e especificidade (E) = 78,9%. A velocidade de sedimentação (VS) apresentou uma AUC ROC = 0,781, ponto de corte = 90,5 mm/h, S = 66,7% e E = 85,7%. O modelo com as duas variáveis apresentou valor p = 0,042 e AUC ROC = 0,790. O modelo Kobayashi apresentou S = 63,6% e E = 77,3%; Egami, S = 66,7% e E = 73,1%; e Sano, S = 28,6% e E = 94,1%.

**Conclusão:**

A PC-R e a VS são variáveis independentes que mostraram tendência preditora de resistência à IGIV com pontos de corte ótimos de 15,1 mg/dL e 90,5 mm/h, respectivamente. Cerca de metade dos pacientes teve algum tipo de envolvimento cardíaco. Os modelos japoneses não têm utilidade nessa população. (Arq Bras Cardiol. 2021; 116(3):485-491)

## Introdução

A doença de Kawasaki (DK) é uma vasculite aguda e autolimitada que afeta os vasos de média dimensão, sendo a principal causa de cardiopatia adquirida em idade pediátrica.^1^

A sua etiologia continua incerta, mas vários fatores têm sido associados à doença, nomeadamente genéticos, ambientais e inflamatórios.^2^ Embora com distribuição mundial, a sua maior prevalência é no Japão, com incidência crescente.^3^ Em Portugal, um estudo epidemiológico realizado em 2017 descreveu uma incidência anual média de 6,5 por 100.000 crianças com menos de 5 anos de idade.^4^

Com base nos critérios da American Pediatric Academy de 2004,^5^ considera-se DK clássica se febre ≥ 5 dias e presença de pelo menos 4 de 5 critérios clínicos adicionais: conjuntivite bilateral não exsudativa, alterações dos lábios e da mucosa oral, exantema polimorfo, alterações das extremidades e linfadenopatia cervical não supurativa. Se febre ≥ 5 dias e apenas 2 ou 3 critérios adicionais, considera-se DK atípica e é necessário recorrer a dados laboratoriais e ecográficos para melhor sustentar o diagnóstico.^2^

Se não tratada atempadamente, a DK pode complicar-se com aneurismas das artérias coronárias (AAC) em até 25% dos casos.^2^ Apesar de o envolvimento coronário ser a consequência mais temida da doença, são possíveis outras complicações cardíacas.^2^^,^^6^^–^^8^ O tratamento com imunoglobulina intravenosa (IGIV) na fase aguda continua a ser a principal terapêutica e, se administrada nos primeiros 10 dias de doença, diminui a incidência de AAC para 4%.^2^ A resistência à IGIV ocorre em 10% a 20% dos casos, aumentado a probabilidade de envolvimento coronário.^2^ Há diferentes abordagens possíveis quando há resistência, nomeadamente uma segunda dose de IGIV, corticosteroides e/ou anticorpos monoclonais.^9^ Não há descrição de benefício na administração inicial de corticosteroide juntamente com a IGIV a todos os pacientes, sendo atualmente tal terapêutica reservada para os casos refratários.^10^ Com o objetivo de identificar os casos que potencialmente poderiam ser resistentes ao tratamento com IGIV, e como tal beneficiar de outras terapêuticas na fase inicial, foram desenvolvidos modelos com base em um sistema de escores, nomeadamente os de Kobayashi,^11^ Egami^12^ e Sano,^13^ que foram validados para a população japonesa. No entanto, diversos estudos mostraram que esses modelos são fracos preditores em diversas populações ocidentais.^10^^,^^14^^,^^15^

O objetivo desse estudo foi identificar fatores preditores clínicos e analíticos de resistência à IGIV e de envolvimento coronário e construir um modelo preditor de resistência mais adequado para essa população. Como objetivos adicionais, pretendeu-se caracterizar os casos de DK em um hospital pediátrico de nível III nos últimos anos, calcular a eficácia dos escores japoneses nessa amostra e analisar as complicações cardíacas não coronárias da DK.

## Métodos

### Amostra

Estudo retrospectivo dos casos diagnosticados com DK desde 1º de janeiro de 2006 a 30 de junho de 2018 no Hospital Pediátrico – Centro Hospitalar e Universitário de Coimbra (HP-CHUC). Foram incluídas todas as crianças e adolescentes com idades entre 30 dias e < 18 anos, com diagnóstico e terapêutica de fase aguda realizados no HP-CHUC. Foram excluídos todos os pacientes transferidos para o HP-CHUC já com diagnóstico e/ou terapêutica realizados em outro hospital.

Foram utilizados os critérios da *American Academy of Pediatrics* para diagnóstico de DK clássica e de DK atípica. Considerou-se D1 de febre o dia inaugural de febre, definida como temperatura axilar ≥ 38°C. Considerou-se resistência à IGIV se a febre persistiu 36 horas após a sua administração, tendo sido excluídos todos os casos em que foi administrado corticosteroide concomitantemente com a primeira dose de IGIV.

Para classificar o envolvimento coronário, foram usados os *escores z* (desvios padrões) de Dallaire, definindo-se dilatação se *escore z* entre 2 e 2,4, aneurisma pequeno se *escore z* entre 2,5 e 4,9, aneurisma médio se entre 5 e 9,9 e dimensão absoluta inferior a 8 mm, aneurisma gigante se *escore z* ≥ 10 ou dimensão absoluta ≥ 8 mm. Nos casos em que a informação foi insuficiente para se calcular o *escore z*, utilizaram-se os valores absolutos, sendo aneurisma pequeno se ≥ 2,5mm e < 4mm, médio se ≥ 4mm e < 8mm e gigante se ≥ 8 mm.

Nas complicações cardíacas, os achados ecocardiográficos de hiperecogenicidade e afunilamento das artérias coronárias não foram considerados.

Para calcular a eficácia dos modelos, foram excluídos todos os pacientes que não apresentavam os dados necessários para serem considerados como alto ou baixo risco de resistência para determinado modelo. A pontuação e a categorização em pacientes de alto ou baixo risco foram realizadas conforme evidenciado na Tabela 1.

**Tabela 1:** Sistema de pontuação dos modelos avaliados

Escore	Pontos	Alto risco
**Kobayashi**		≥ 4 pontos
AST > 100 U/L	2	
Na ≤ 133 mmol/L	2	
IGIV com febre ≤ D4	2	
Neutrófilos/Leucócitos ≥ 80%	2	
PC-R ≥ 10 mg/dL	1	
Idade ≤ 1 ano	1	
Plaquetas ≤ 300.000/uL	1	
**Egami**		≥ 3 pontos
ALT ≥ 80 U/L	2	
IGIV com febre ≤ D4	1	
PC-R ≥ 8 mg/dL	1	
Idade ≤ 6 meses	1	
Plaquetas ≤ 300.000/uL	1	
**Sano**		≥ 2 pontos
AST > 200 U/L	1	
Bilirrubina total ≥ 0,9 mg/dL	1	
PC-R ≥ 7 mg/dL	1	

ALT: alanina aminotransferase; AST: aspartato aminotransferase; dL: decilitro; L: litro; mg: miligrama; IGIV: imunoglobulina intravenosa; mmol: milimole; Na: sódio plasmático; PC-R: proteína C-reativa; U: unidade internacional; uL: microlitro.

### Análise Estatística

Análise estatística efetuada com recurso ao programa SPSS® (IBM®, SPSS® Statistics Inc., Chicago), versão 25.0. Para testar a normalidade da amostra, foi utilizado o teste de Shapiro-Wilk. As variáveis contínuas com distribuição normal foram descritas por meio de média e desvio padrão, as variáveis contínuas sem distribuição normal foram descritas através de mediana e amplitude interquartil (AIQ). Para comparação de variáveis categóricas, foi utilizado o teste exato de Fisher; das variáveis numéricas paramétricas, o teste de t-student não pareado; e das variáveis numéricas não paramétricas, o teste de Mann-Whitney. Foram construídas curvas *receiver operating characteristic* (ROC) para avaliar a capacidade discriminativa individual de cada variável e identificar pontos de corte para a predição de resistência à IGIV. As variáveis foram consideradas preditoras se com uma *area under the curve* (AUC) superior a 0,75. Para a elaboração de um modelo preditor de resistência, foi utilizada a regressão logística multivariada. O nível de significância utilizado nesse estudo foi de 5%.

## Resultados

Cumpriram critérios de DK 48 pacientes, sendo 32 (66,7%) do sexo masculino. A mediana de idades foi de 36 meses (AIQ 16,75-89,25), com 62,5% abaixo dos 5 anos de idade e 10,4% acima dos 9 anos. No dia de admissão, a totalidade dos pacientes apresentava-se com febre, com uma mediana de 5 dias de febre (AIQ 4-8), mínimo de 1 e máximo de 14 dias. Dos cinco critérios clínicos principais, observou-se conjuntivite não purulenta em 94%, alterações orais em 90%, exantema em 84%, alterações das extremidades em 75% e linfadenopatia cervical em 69%. Dentre as alterações orais, as mais prevalentes foram a queilite (67%) e o eritema labial (67%), seguidos de eritema da orofaringe (50%) e língua em framboesa (48%). Entre as alterações das extremidades, a mais prevalente foi eritema (52%), seguido de edema duro (31%) e descamação (25%). Os sinais inflamatórios no local de inoculação da vacina *Bacillus Calmette-Guérin* (BCG) foram descritos em 23%. Foi diagnosticada DK atípica em 17% dos casos. A mediana de dias de internamento foi de 2 dias (AIQ 1-6,75).

A totalidade dos pacientes foi medicada em fase aguda com IGIV 2 g/kg com mediana do dia de administração de 6,5 dias (AIQ 5-8). Foram medicados em fase aguda 47 pacientes com ácido acetilsalicílico (AAS) em doses entre 45 a 100mg/kg/dia. Cinco pacientes foram ainda medicados com corticosteroide juntamente com a primeira dose de IGIV. Após a fase aguda, todos os pacientes foram medicados com AAS em dose de 3 a 5 mg/kg/dia, três pacientes com clopidogrel e um com enoxaparina. Verificou-se resistência à IGIV em nove casos (21%), dos quais havia um caso de DK atípica (p = 0,543). Dos nove pacientes resistentes à terapêutica, foi administrada uma segunda dose de IGIV 2 g/kg e a cinco destes, metilprednisolona na dose de 30 mg/kg/dia. 

Entre as diferentes variáveis avaliadas como preditoras de resistência (Tabela 2), a proteína C-reativa (PC-R) apresentou uma AUC ROC de 0,78 (IC95%: 0,632-0,947) e a velocidade de sedimentação (VS) uma AUC ROC de 0,781 (IC95%: 0,585-0,977). O ponto de corte para a PC-R foi de 15,1 mg/dL com uma sensibilidade (S) de 0,778 e especificidade (E) de 0,789 [*Odds ratio* (OR) = 13,125 IC95%: 2,271-75,858]. O ponto de corte para a VS foi de 90,5 mm/h, verificando-se uma sensibilidade de 0,667 e especificidade de 0,857 (OR = 12,000 IC95%: 1,718-83,803). Foi ajustado um modelo logístico com as duas variáveis PC-R e VS, modelo este que apresentou um valor p de 0,042 e AUC ROC de 0,790 (IC95%: 0,589-0,992). No ponto de corte ótimo, a sensibilidade foi de 0,833 e a especificidade foi de 0,771. Apresentou ainda uma variância de 25% (Nagelkerke R2 = 0,254).

**Tabela 2:** Análise ROC de diversas variáveis para predizer resistência à IGIV

Variável	AUC [IC 95%]
Idade	0,542 [0,377; 0,708]
Dia de administração IGIV	0,595 [0,403; 0,787]
Hemoglobina	0,611 [0,416; 0,806]
Leucócitos	0,525 [0,331; 0,719]
Neutrófilos	0,637 [0,447; 0,828]
Plaquetas	0,513 [0,295; 0,732]
VS	0,781 [0.585; 0,977]
PC-R	0,789 [0,632; 0,947]
Na	0,715 [0,475; 0,955]
AST	0,648 [0,434; 0,862]
ALT	0,693 [0,486; 0,901]
Bilirrubina total	0,500 [0,139; 0,861]
Albumina	0,693 [0,459; 0,928]

ALT: alanina aminotransferase; AST: aspartato aminotransferase; AUC: área abaixo da curva; IC: intervalo de confiança; IGIV: imunoglobulina intravenosa; Na: sódio plasmático; PC-R: proteína C-reativa; ROC: receiver operating curve; VS: velocidade de sedimentação.

Ocorreu envolvimento coronário em 12 casos (25%). Sete pacientes desenvolveram dilatação das artérias coronárias e cinco, AAC. Na Tabela 3, comparam-se os grupos sem e com envolvimento coronário. Observaram-se diferenças apenas na duração da febre e na utilização de corticosteroides. O tempo total de febre foi mais elevado no grupo com envolvimento coronário (p = 0,038). Esse grupo recebeu mais vezes corticosteroide (p = 0,009). Quatro desses pacientes já tinham diagnóstico de envolvimento coronário prévio à administração desse fármaco. Dos pacientes com AAC, três cumpriram critérios para aneurismas pequenos, um para aneurismas médios e um para aneurismas gigantes. Esses pacientes estão caracterizados na Tabela 4.

**Tabela 3:** Características dos grupos com e sem envolvimento coronário

Variáveis	Sem envolvimento coronário (n =36)	Envolvimento coronário (n = 12)	p
Corticosteroide (n = 10) N	4	6	0,009
Resistência à IGIV (n = 9) N	5	4	0,173
Idade (meses) Média ± DP	59,7 ± 57	47,5 ± 30,1	0,35
Dia de resolução da febre Média ± DP	7,4 ± 2,8	9,4 ± 3	0,038
Dia de administração de IGIV Média ± DP	6,5 ± 2,9	7,6 ± 3,5	0,283
DK atípica n	6	2	0,686
Hemoglobina (g/dL) Média ± DP	11,5 ± 1,3	11,2 ± 1,3	0,359
Leucócitos (/uL) Média ± DP	14.174 ± 6.010	15.216 ± 6.918	0,619
Neutrófilos (/uL) Média ± DP	9.515 ± 4.770	10.994 ± 5.629	0,378
Plaquetas (/uL) Média ± DP	32.8389 ± 12.7125	36.2667 ± 28.2652	0,691
PC-R (mg/dL) Média ± DP	10,4 ± 8,8	19,6 ±25	0,243
VS (mm/h) Média ± DP	71,6 ± 19	76,7 ± 33,4	0,672
Na (mmol/L) Média ± DP	137 ± 4	137 ± 5	0,869
AST (U/L) Média ± DP	64 ± 53	101 ± 91	0,222
ALT (U/L) Média ± DP	99 ± 116	113 ± 86	0,712
Bilirrubina total (mg/dL) Média ± DP	1,9 ± 2	2,8 ± 3	0,538
Albumina (g/L) Média ± DP	35,6 ± 4,3	34,9 ± 7,2	0,77

AIQ: amplitude interquartil; ALT: alanina aminotransferase; AST: aspartato aminotransferase; dL: decilitro; DK: doença de Kawasaki; DP: desvio padrão; g: grama; h: hora; IGIV: imunoglobulina intravenosa; L: litro; mg: miligrama; mmol: milimole; n: número absoluto; Na: sódio plasmático; p: valor p; PC-R: proteína C-reativa; U: unidade internacional; uL: microlitro; VS: velocidade de sedimentação.

**Tabela 4:** Características dos pacientes com aneurismas das artérias coronárias

Variáveis	Paciente 1	Paciente 2	Paciente 3	Paciente 4	Paciente 5
Idade de diagnóstico (meses)	12	60	108	63	4
Sexo masculino	Sim	Sim	Sim	Sim	Sim
Dias de febre	7	6	8	9	14
DK clássico	Sim	Sim	Sim	Sim	Não
Dia de febre na 1ᵃ dose IGIV	7	6	4	6	14
Resistência ao tratamento IGIV	Não	Não	Sim	Sim	NA
Dia de febre na 2ᵃ dose IGIV	NA	NA	6	8	NA
MPDN (30mg/kg/dia) Com 1ᵃ dose IGIV Com 2ᵃ dose IGIV	Não NA NA	Não NA NA	Não Não Sim	Sim Não Sim	Sim Sim Não
Classificação AAC	Pequenos	Pequenos	Pequenos	Médios	Gigantes
Z escore máximo	NA	4,46	3,56	6,94	13,81
Artérias atingidas	ACD; TC	ACD; ACE	ACE	ACE; ACD	ACD; CIR; DAE

AAC: aneurismas das artérias coronárias; ACD: artéria coronária direita; ACE: artéria coronária esquerda; CIR: artéria circunflexa; DAE: descendente anterior esquerda; DK: doença de Kawasaki; IGIV: imunoglobulina intravenosa; MPDN: metilprednisolona; NA: não aplicável; TC: tronco comum.

Na fase aguda, para além do envolvimento coronário, observou-se derrame pericárdico em 10 casos, insuficiência valvular mitral ligeira em três e disfunção ventricular em três, um dos quais com choque cardiogênico. Um apresentou bloqueio atrioventricular (BAV) de 1ºgrau variável. Após a fase aguda, um paciente manteve envolvimento do sistema de condução e dilatação do ventrículo esquerdo (VE) e um ficou com hipertrofia do VE.

Os valores de S, E, valor preditivo positivo (VPP) e valor preditivo negativo (VPN) para os diferentes modelos estão apresentados na tabela 5, sendo apenas incluídos os pacientes a quem foi possível categorizar com alto ou baixo risco.

**Tabela 5:** Sensibilidade, especificidade, valores preditivos positivos e negativos dos diferentes modelos

Modelo	n	S	E	VPP	VPN
Kobayasahi	34	63,6%	77,3%	53,8%	81%
Egami	39	66,7%	73,1%	50%	82,6%
Sano	25	28,6%	94,1%	66,7%	77,3%

E: especificidade; n: número de casos incluídos; S: sensibilidade; VPN: valor preditivo negativo; VPP: valor preditivo positivo.

## Discussão

A DK é uma vasculite que, embora não tendo uma incidência tão elevada como no Japão, é, ainda assim uma causa importante de doença em idade pediátrica na nossa população. Um diagnóstico e introdução de terapêutica precoces constituem dois fatores muito importantes para reduzir o risco de envolvimento cardíaco.

O presente estudo revelou uma percentagem de casos resistentes à IGIV coincidentes com os 10 a 20% descritos na literatura.^2^ Ao longo dos anos têm sido desenvolvidos esforços no sentido de encontrar fatores clínicos e laboratoriais que possam prever esta resistência de modo a introduzir mais precocemente terapêuticas coadjuvantes. Existem diversos parâmetros descritos na literatura tais como idade, albumina, transaminases, bilirrubina total, neutrófilos, plaquetas, PC-R, VS, entre outros.^14^^,^^16^^–^^20^ Neste estudo observou-se que a PC-R e a VS apresentaram capacidade preditora estatisticamente significativa de resistência à IGIV. No caso da PC-R, o ponto de corte ótimo foi de 15,1 mg/dL, com uma sensibilidade de 0,778, especificidade de 0,789 e um OR de 13,125. Pacientes com PC-R superior a 15,1mg/dL apresentam uma probabilidade de resistência à IGIV cerca de 13 vezes superior aos que têm valores inferiores. Relativamente à VS, o ponto de corte ótimo foi de 90,5 mm/h, com uma sensibilidade de 0,667, especificidade 0,857 e OR 12,000. Pacientes com VS superior a 90,5 mm/h apresentam uma probabilidade de resistência à IGIV cerca de 12 vezes superior aos que têm valores inferiores. Combinando estas duas variáveis independentes obteve-se um modelo estatisticamente significativo (p = 0,042), cujo ponto de corte apresenta uma sensibilidade de 0,833 e especificidade de 0,771. No entanto, a variância explicada pelo modelo é apenas de 25% (Nagelkerke R2 = 0,254), pelo que, embora seja estatisticamente significativo, não pode ser validado, o que se deve em grande parte ao tamanho reduzido da amostra. Todavia, é importante realçar esta tendência no sentido de que as duas variáveis poderão ser importantes para prever a resistência à IGIV, antes da sua infusão. O fato destas variáveis terem sido preditoras de resistência, realça o papel da inflamação exuberante nesta doença, etiologia que é defendida como possível precipitante da resposta imunológica que culmina numa vasculite.^21^

A etiologia da DK continua incerta, no entanto vários fatores são apontados como predisponentes. Um deles é a imaturidade do sistema imunitário, condição que é apoiada pelo fato de afetar predominantemente crianças com idade inferior a cinco anos. Neste estudo 62,5% dos pacientes pertenciam a esta faixa etária, o que, embora corresponda à maioria da amostra, fica aquém dos 80% descritos na literatura.^1^ Uma possível explicação para estes resultados é a contribuição genética, sendo as percentagens reportadas baseadas em estudos com variedade étnica alargada, incluindo asiáticos.

Verificou-se envolvimento coronário em 25% dos casos, com sete a cumprir critérios de dilatação e cinco de aneurismas das artérias coronárias. Os 10% de incidência de AAC, foram superiores aos 4% reportados para os casos devidamente tratados. Comparando os grupos com e sem envolvimento coronário, verificou-se diferença estatisticamente significativa relativamente ao tempo total de febre (p = 0,038). Este resultado corrobora a ideia da persistência da febre ser deletéria e a necessidade da administração de IGIV preferencialmente até ao 10º dia para evitar sequelas cardíacas.^9^ O uso de corticosteróide na DK continua a ser tema de debate e controvérsia. O mais consensual é usar metilprednisolona por via intravenosa (MPDN iv) na dose de 15 a 30mg/kg/dia durante três dias.^9^ A MPDN iv, nos pacientes com DK refratária à IGIV, suprime os níveis de citocinas inflamatórias mais rapidamente que uma 2ᵃ dose de IGIV,^9^ no entanto não está recomendada como primeira linha. Sleeper et al.^10^ avaliaram o uso de corticosteróide em diferentes momentos, observando-se diferença estatisticamente significativa no aparecimento de AAC apenas nos pacientes refratários à IGIV e que levaram a administração de segunda dose.

Neste estudo também se avaliaram complicações cardíacas e achados ecográficos que não o envolvimento coronário. Das complicações cardíacas em fase aguda mais graves destacaram-se três casos de disfunção ventricular esquerda, um com choque cardiogénico e um BAV de 1º grau, complicações essas também descritas na literatura.^2^^,^^7^ Na fase aguda, foi ainda possível observar 10 pacientes com derrame pericárdico sem compromisso hemodinâmico e três com insuficiência valvular mitral ligeira. Em Paris, Chbeir et al.^22^ obtiveram relação estatisticamente significativa entre resistência à IGIV, AAC e achados na ecografia cardíaca inicial, tais como derrame pericárdico, hiperecogenicidade das coronárias e dilatação coronária. Os autores desse estudo não consideraram a hiperecogenicidade e o afunilamento das coronárias como fatores relevantes, visto que são achados subjetivos, pouco reprodutíveis e podem ser encontrados tanto em doenças febris como em crianças saudáveis.^23^ Na fase crônica, um paciente manteve envolvimento do sistema de condução e dilatação do VE e outro ficou com hipertrofia do VE. As repercussões cardíacas a longo prazo na DK estão ainda pouco esclarecidas. Friedman et al.^6^ mostraram maior ocorrência de efeitos adversos cardíacos a longo prazo, tais como morte, transplante cardíaco, cirurgias de *bypass* coronário e angioplastia primária em pacientes com DK que desenvolveram AAC com *escores z* superiores e que foram resistentes à IGIV. Um estudo realizado por Holve et al.^8^ revelou baixa incidência de efeitos adversos cardíacos até os 21 anos de idade, mas uma maior probabilidade de desenvolver hipertensão arterial após os 15 anos de idade.

Os modelos validados no Japão apresentaram fraca utilidade clínica no presente estudo (Tabela 4). O modelo com maior especificidade foi o de Sano, embora com sensibilidade muito baixa e apenas com um pequeno número de casos incluído. O modelo de Egami foi o mais sensível para esta amostra, mas, ainda assim, insuficiente para ser validado. Na base dessa diferença de resultados, pode estar o componente genético. Aliás esses resultados vão ao encontro de outros trabalhos realizados fora do Japão, em que nenhum deles conseguiu validar os modelos nas suas amostras.^10^^,^^14^^–^^17^^,^^20^ É necessário levar em consideração ainda que existiram algumas diferenças no desenho do estudo relativamente aos modelos japoneses, nomeadamente aplicados apenas a pacientes com DK clássica. Um estudo japonês falhou em validar os modelos em uma amostra de pacientes apenas com DK atípica.^24^ O modelo de Kobayashi foi validado na população japonesa com uma sensibilidade de 0,86 e especificidade de 0,67.^11^ Ao contrário do presente estudo, a IGIV foi administrada na dose de 1 g/kg em dois dias consecutivos e foi considerada resistência se febre persistente após 24 horas de início de terapêutica, ou se recrudescência com sintomas após período apirético. O modelo de Egami foi validado com uma sensibilidade de 0,78 e especificidade de 0,76; no entanto, foi definida resistência se ausência de diminuição do valor de PC-R em mais de 50% e persistência da febre 48 horas após administração de IGIV.^12^ Loomba et al.^25^ não conseguiram validar o modelo de Egami, mesmo aplicando-o separadamente à DK clássica, atípica e por etnias. O modelo de Sano, validado com uma sensibilidade de 0,77 e especificidade de 0,86, foi o único dos três a ajustar o tamanho dos AAC à superfície corporal.^13^ No entanto, também usou administração de IGIV na dose de 1 g/kg em 2 dias consecutivos e definiu resistência se persistência de febre após 24 horas do término da terapêutica.

Este trabalho apresenta as limitações inerentes ao fato de ser um estudo retrospectivo e com uma amostra de tamanho reduzido.

## Conclusão

A PC-R e a VS são variáveis independentes que mostraram tendência preditora de resistência à IGIV com pontos de corte ótimos de 15,1 mg/dL e 90,5 mm/h, respectivamente. Existe, no entanto, necessidade de um estudo com uma amostra de dimensões adequadas para validar um modelo baseado nesses dois dados analíticos. As complicações cardíacas não se resumem às artérias coronárias, devendo ser mais abrangentes o estudo e o seguimento desses pacientes. Os modelos validados para a população japonesa apresentam utilidade muito limitada na amostra deste estudo, reforçando ainda mais a necessidade e a importância de novas abordagens.
