# Supplementary material for: Doença de Kawasaki: Preditores de Resistência à Imunoglobulina Intravenosa e Complicações Cardíacas
Source: Arq Bras Cardiol. 2021 Mar 3;116(3):485–91. [Article in Portuguese] doi: 10.36660/abc.20190758 (PMC8159558; doi:10.36660/abc.20190758)
Supplement: Translation

# Translation: Kawasaki Disease: Predictors of Resistance to Intravenous Immunoglobulin and Cardiac Complications

**Authors:** Diogo Faim, Cláudio Henriques, Ana Brett, Andreia Francisco, Fernanda Rodrigues, António Pires

**Affiliations:** 1 Coimbra Portugal Centro Hospitalar e Universitário de Coimbra EPE - Cardiologia Pediátrica Coimbra - Portugal; 2 Coimbra Portugal Centro Hospitalar e Universitário de Coimbra EPE - Urgência e Unidade de Infeciologia, Coimbra - Portugal

**Keywords:** Kawasaki Disease/complications, Mucocutaneous Lymph Node Syndrome/complications, Drug Resistance, Coronary Artery Disease, Immunoglobulin, Child

## Abstract

**Background:**

Kawasaki disease (KD) is the leading cause of acquired cardiac disease in children, in developed countries.

**Objectives:**

To identify predictive factors for resistance to intravenous immunoglobulin (IVIG), calculate the effectiveness of Japanese predictive models and characterize cardiac complications.

**Methods:**

Retrospective analysis of KD cases admitted in a Portuguese paediatric hospital between january 2006 and july 2018. ROC curves were used to determine predictive factors for resistance and the multivariate logistic regression analysis was used to develop the predictive model. A significance level of 5% was used.

**Results:**

48 patients with a median age of 36 months were included. The IVIG resistance was 21%. Echocardiographic anomalies were noted in 46%, with coronary involvement in 25% of the sample population. As predictive variable of resistance, the C-reactive protein (CRP) presented an AUC ROC = 0.789, optimal cut-off value 15.1 mg/dL, sensitivity (Sn) 77.8% and specificity (Sp) 78.9%. The erythrocyte sedimentation rate (ESR) presented an AUC ROC = 0.781, optimal cut-off value 90.5 mm/h, Sn 66.7% and Sp 85.7%. The model with the two variables showed p = 0.042 and AUC ROC = 0.790. Predictive strength of Japanese models were: Kobayashi (Sn 63.6%, Sp 77.3%), Egami (Sn 66.7%, Sp 73.1%), Sano (Sn 28.6%, Sp 94.1%).

**Conclusion:**

CRP and ESR are independent variables that were related to IVIG resistance, with optimal cut-off points of 15.1 mg/dL and 90.5 mm/h, respectively. About half of the patients had some form of cardiac involvement. The Japanese models appeared to be inadequate in our population. (Arq Bras Cardiol. 2021; 116(3):485-491)

## Introduction

Kawasaki disease (KD) is an acute self-limiting vasculitis, which affects medium-sized vessels and is the leading cause of acquired cardiac disease in pediatric age groups.^1^

Its etiology remains uncertain, but several factors have been associated to it, namely genetic, environmental, and inflammatory ones.^2^ Although with a worldwide distribution, its highest prevalence is in Japan, where the incidence is on the rise.^3^ In Portugal, an epidemiological study carried out in 2017 showed an mean annual incidence of 6.5 per 100,000 children under 5 years of age.^4^

Based on the 2004 American Pediatric Academy criteria,^5^ classic KD is considered if fever persists for five days or more and if at least four of five additional clinical criteria are observed: nonexudative bilateral conjunctivitis, alterations of the lips and oral cavity, erythematous rash, changes in the extremities, and cervical lymphadenopathy. If fever lasts for five or more days and only two or three additional criteria are present, it is considered atypical KD, if supported by laboratory and echocardiographic data.^2^

If not treated within an established period, KD can be complicated by coronary artery aneurysms (CAA) in up to 25% of cases.^2^ Although coronary artery involvement is the most feared consequence of the disease, other cardiac complications are possible.^2^^,^^6^^–^^8^ Treatment with intravenous immunoglobulin (IVIG) in the acute phase administered in the first 10 days of illness reduces the incidence of CAA to 4%.^2^ IVIG resistance occurs in 10-20% of cases, increasing the likelihood of coronary involvement.^2^ There are different possible approaches in case of IVIG resistance, such as a second dose of IVIG, corticosteroids and/or monoclonal antibodies.^9^ No benefit has been described when corticosteroids are used in addition to IVIG in the first instance and this therapeutics is currently reserved for refractory cases.^10^ In order to identify the cases that could potentially be resistant to treatment with IVIG, and benefit from adjuvant therapies in the initial phase, models based on a scoring system have been developed. Three have been validated in the Japanese population, namely the Kobayashi,^11^ Egami,^12^ and Sano scoring systems.^13^ However, several studies have shown that these models are poor predictors in many western populations.^10^^,^^14^^,^^15^

The aim of this study was to identify clinical and laboratory predictive factors regarding resistance to IVIG and coronary artery involvement, and to develop a more suitable predictor model of resistance in this population. Secondary objectives regard characterizing the KD cases admitted to a central pediatric hospital over a period of 13 years, to verify the effectiveness of the Japanese scoring systems in our population sample and to analyze the non-coronary cardiac complications of KD.

## Methods

### Sample

Retrospective analysis of KD cases admitted to the Hospital Pediátrico – Centro Hospitalar e Universitário de Coimbra (HP-CHUC) diagnosed from 01/01/2006 to 06/30/2018. All patients between 30 days and <18 years with KD and treated with IVIG at diagnosis at HP-CHUC were included in the study. All the patients transferred from outlying centers with a diagnosis of KD and managed at these institutions were excluded.

The diagnosis of typical and atypical KD was based on the American Academy of Pediatrics criteria. We considered day one of fever on the day the fever started, defined as axillar temperature ≥ 38ºC.

Resistance to IVIG was considered if the fever persisted 36 hours after its administration. All patients who received corticosteroids simultaneously with the first dose of IVIG were excluded from the resistance quantification.

Dallaire z scores were used to classify the coronary artery morphology defining: coronary artery ectasia if z score between 2 and 2.4, small aneurysm if z score between 2.5 and 4.9, medium aneurysm if z score between 5 and 9.9 and absolute dimension < 8 mm, and giant aneurysm if z score ≥ 10 or absolute dimension ≥ 8 mm. If unable to calculate the z score, the absolute dimensions were used, being small aneurysm if ≥ 2.5 mm and < 4mm, medium aneurysm if ≥ 4 mm and < 8 mm and giant aneurysm if ≥ 8 mm.

Regarding other cardiac complications, the coronary artery hyperechogenicity and lack of tapering on echocardiography were not considered as echocardiographic diagnostic criteria.

To calculate the effectiveness of the Japanese models, all patients who did not have the necessary data to be considered as high or low risk of IVIG resistance were excluded. Scoring and categorization in high or low risk patients were performed as shown in Table 1.

**Table 1:** Scoring system of the Japanese modelss

Model	Score	High risk
**Kobayashi**		≥ 4 points
AST > 100 U/L	2	
Na ≤ 133 mmol/L	2	
IVIG with fever ≤ 4 days	2	
Neutrophils/Leucocytes ≥ 80%	2	
CRP ≥ 10 mg/dL	1	
Age ≤ 1 year old	1	
Platelets ≤ 300,000/µL	1	
**Egami**		≥ 3 points
ALT ≥ 80 U/L	2	
IVIG with fever ≤ 4 days	1	
CRP ≥ 8 mg/dL	1	
Age ≤ 6 months	1	
Platelets ≤ 300,000/µL	1	
**Sano**		≥ 2 points
AST > 200 U/L	1	
Total bilirubin ≥ 0.9 mg/dL	1	
CRP ≥ 7 mg/dL	1	

AST: aspartate transaminase; U: international unity; L: liter; Na: serum sodium; mmol: millimole; IVIG: intravenous immunoglobulin; CRP: C-reactive protein; mg: milligram; dL: deciliter; µL: microliter; ALT: alanine transaminase.

### Statistical Analysis

The SPSS® (IBM®, SPSS® Statistics Inc., Chicago) program version 25.0 was used the performed the statistical analysis. The Shapiro-Wilk test was used to test the normality of the variables. The continuous variables with normal distribution were described using mean and standard deviation (SD) and continuous variables without normal distribution were described using median and interquartile range (IQR). We used the Fisher’s exact test to compare categorical variables, the Student’s t-test to compare parametric variables and the Mann-Whitney test to compare the non-parametric ones. The Receiver Operating Characteristic (ROC) curves were used to evaluate the individual discriminative capacity of each variable and to identify the optimal cutoff points to predict resistance to IVIG. The variables were considered as good predictors if the area under the curve (AUC) > 0.75. Multivariate logistic regression analysis was used to develop the predictive resistance model. A significance level of 5% was used.

## Results

Forty-eight patients met the KD criteria, of whom 32 (66.7%) were male. The median age was 36 months (IQR 16.75-89.25), 62.5% of patients were less than five years old and 10.4% over nine years old. On the day of admission, all the patients presented with fever, with a median duration of five days (IQR 4-8), minimum of one day and maximum of 14 days. Among the five main clinical criteria, nonexudative bilateral conjunctivitis was observed in 94% of cases, alterations of the lips and oral cavity in 90%, erythematous rash in 84%, changes in extremities in 75%, and cervical lymphadenopathy in 69%. The most common findings among oral alterations were cheilitis (67%) and lip erythema (67%), followed by erythema of the oropharynx (50%) and the strawberry tongue (48%). The most prevalent changes in the extremities were erythema (52%), followed by swelling (31%) and peeling (25%). Inflammatory signs at the Bacillus Calmette-Guérin (BCG) vaccination site were observed in 23% of patients. Atypical KD was diagnosed in 17% of the cases. The median duration of hospital stay was two days (IQR 1-6.75).

During the acute phase, IVIG 2g/kg was administered to all patients. The median day of illness of the administration was 6.5 (IQR 5-8). Simultaneously, 45-100mg/kg of acetylsalicylic acid (ASA) were administered to 47 patients in the acute phase. Five children received corticosteroids together with the first dose of IVIG. After the acute phase, all patients were medicated with ASA 3-5 mg/kg/day, three patients with clopidogrel and one with enoxaparin. Nine patients were resistant to the IVIG (21%), of which one had atypical KD (p = 0.543). All nine repeated IVIG administration, five of which with methylprednisolone 30 mg/kg/day.

Among the variables evaluated as predictors of IVIG resistance (Table 2), C-reactive protein (CRP) presented an AUC ROC of 0.78 (95% confidence interval (CI): 0.632 – 0.947), and the erythrocyte sedimentation rate (ESR), an AUC ROC of 0.781 (95%CI: 0.585 – 0.977). The optimal cut-off value for CRP was 15.1 mg/dL with sensitivity (Sn) of 77.8% and specificity (Sp) of 78.9% (Odds ratio (OR) = 13.125 95%CI: 2.271 – 75.858). The optimal cut-off value for ESR was 90.5 mm/h, with Sn of 66.7% and Sp of 85.7% (OR = 12.000 95%CI: 1.718 – 83.803). A logistic model was developed with these two variables, with a p-value of 0.042, AUC ROC of 0.79 (95%CI: 0.589 – 0.992), with Sn of 83.3% and Sp of 77.1%, but with a 25% variance (Nagelkerke R2 = 0.254).

**Table 2:** Receiver operating characteristic analysis of several variables to predict resistance to intravenous hemoglobulin

Characteristic	AUC [95%CI]
Age	0.542 [0.377; 0.708]
IVIG administration day	0.595 [0.403; 0.787]
Hemoglobin	0.611 [0.416; 0.806]
Leucocytes	0.525 [0.331; 0.719]
Neutrophils	0.637 [0.447; 0.828]
Platelets	0.513 [0.295; 0.732]
ESR	0.781 [0.585; 0.977]
CRP	0.789 [0.632; 0.947]
Na	0.715 [0.475; 0.955]
AST	0.648 [0.434; 0.862]
ALT	0.693 [0.486; 0.901]
Total bilirubin	0.500 [0,139; 0.861]
Albumin	0.693 [0.459; 0.928]

AUC: area under the curve; CI: confidence interval; IVIG: intravenous immunoglobulin; ESR: erythrocyte sedimentation rate; CRP: C-reactive protein; Na: serum sodium; AST: aspartate transaminase; ALT: alanine transaminase.

Coronary artery changes were found in 12 children (25%), seven with ectasia and five with CAA. The comparison between groups with and without coronary artery involvement is shown in Table 3. The duration of fever and the use of corticosteroids were the only significant differences between these two groups. Patients with coronary artery involvement had longer duration of fever (p = 0.038) and greater need for corticotherapy (p = 0.009). Four patients had CAA when methylprednisolone was started. Among the five patients with CAA, three met the criteria for small aneurysms, one for medium aneurysms and one for giant ones. These patients are summarized in Table 4.

**Table 3:** Characteristics of groups with and without coronary artery involvement

Characteristics	Without coronary involvement (n =36)	Coronary involvement (n= 12)	p - value
Corticoid (n=10) n	4	6	0.009
Resistance to IVIG (n=9) n	5	4	0.173
Age (months) Mean ± sd	59.7 ± 57	47.5 ± 30.1	0.35
Fever duration (days) Mean ± sd	7.4 ± 2.8	9.4 ± 3	0.038
IVIG administration day Mean ± sd	6.5 ± 2.9	7.6 ± 3.5	0.283
Atypical KD n	6	2	0.686
Haemoglobin (g/dL) Mean ± sd	11.5 ± 1.3	11.2 ± 1.3	0.359
Leucocytes (/µL) Mean ± sd	14,174 ± 6,010	15,216 ± 6,918	0.619
Neutrophils (/µL) Mean ± sd	9,515 ± 4,770	10,994 ± 5,629	0.378
Platelets (/µL) Mean ± sd	328,389 ± 127,125	362,667 ± 282,652	0.691
CRP (mg/dL) Mean ± sd	10.4 ± 8.8	19.6 ±25	0.243
ESR (mm/h) Mean ± sd	71.6 ± 19	76.7 ± 33.4	0.672
Na (mmol/L) Mean ± sd	137 ± 4	137 ± 5	0.869
AST (U/L) Mean ± sd	64 ± 53	101 ± 91	0.222
ALT (U/L) Mean ± sd	99 ± 116	113 ± 86	0.712
Total bilirubin (mg/dL) Mean ± sd	1.9 ± 2	2.8 ± 3	0.538
Albumin (g/L) Mean ± sd	35.6 ± 4.3	34.9 ± 7.2	0.77

n: absolute value; IVIG: intravenous immunoglobulin; sd: standard deviation; KD: Kawasaki disease; g: gram; dL: deciliter; µL: microliter; CRP: C-reactive protein; mg: milligram; ESR: erythrocyte sedimentation rate; h: hour; Na: serum sodium; mmol: millimole; L: litre; AST: aspartate transaminase; U: international unity; ALT: alanine transaminase.

**Table 4:** Characteristics of patients with coronary artery aneurysms

Characteristics	Patient 1	Patient 2	Patient 3	Patient 4	Patient 5
Age (months)	12	60	108	63	4
Male	Yes	Yes	Yes	Yes	Yes
Fever duration (days)	7	6	8	9	14
Classic KD	Yes	Yes	Yes	Yes	No
Day of fever in IVIG 1^st^ dose	7	6	4	6	14
Resistance to IVIG	No	No	Yes	Yes	NA
Day of fever in IVIG 2^nd^ dose	NA	NA	6	8	NA
MPDN (30mg/Kg/day) With IVIG 1^st^ dose With IVIG 2^nd^ dose	No NA NA	No NA NA	Yes No Yes	Yes No Yes	Yes Yes No
CAA classification	Small	Small	Small	Medium	Giant
Maximum z score	NA	4.46	3.56	6.94	13.81
Arteries involved	RCA; CT	RCA; LCA	LCA	RCA; LCA	RCA; LAD; LCX

KD: Kawasaki disease; IVIG: intravenous immunoglobulin; MPDN: methylprednisolone; mg: milligram; Kg: kilogram; CAA: coronary artery aneurysms; RCA: right coronary artery, CT: common trunk; LCA: left coronary artery; LAD: left anterior descending artery; NA: not applicable; LCX: left circumflex artery.

In the acute phase, in addition to coronary involvement, 10 patients presented with pericardial effusion, three with mild mitral valve regurgitation, two with left ventricular systolic dysfunction, one with cardiogenic shock and one with variable first degree atrioventricular (AV) block. After the acute phase, the patient with the AV block developed left ventricle (LV) dilation and another patient developed LV hypertrophy.

Table 5 summarizes Sn, Sp,and the positive (PPV) and negative predictive values (NPV) for the Japanese models in our sample.

**Table 5:** Statistical values of the Japanese models in our study

Model	n	Sn (%)	Sp (%)	PPV (%)	NPV (%)
Kobayasahi	34	63.6	77.3	53.8	81
Egami	39	66.7	73.1	50	82.6
Sano	25	28.6	94.1	66.7	77.3

n: absolute value; Sn: sensitivity; Sp: Specificity; PPV: positive predictive value; NPV: negative predictive value.

## Discussion

Despite the lower incidence compared to Japan, KD is a vasculitis that is still an important cause of pediatric disease in our population. Early diagnosis and management are two important factors that appear to reduce cardiac involvement.

Our study revealed an incidence of IVIG resistance similar to the 10 to 20% described in the literature.^2^ Over the years, efforts have been made to find clinical and laboratory factors that can predict this resistance in order to introduce adjuvant therapies at an early stage of the disease. There are several parameters in the literature that have been studied for this purpose, such as age, serum albumin, transaminases, total bilirubin, neutrophils count, platelet count, CRP, ESR, among others.^14^^,^^16^^–^^20^ In our study, CRP and ESR presented a statically significant predictive capacity in relation to IVIG resistance. For CRP, the optimal cut-off point was 15.1 mg/dL (Sn 77.8%, Sp 78.9%, OR 13.125). Patients with CRP values above 15.1 mg/dL are about 13 times more likely to be resistant to IVIG than those with lower values. Concerning ESR, the optimal cut-off point was 90.5 mm/h (Sn 66.7%, Sp 85.7%, OR 12.000). Patients with ESR greater than 90.5 mm/h have a probability of resistance to IVIG approximately 12 times higher than those with lower values. Combining these two independent variables, a statistically significant model was obtained (p = 0.042), whose cut-off point has Sn of 83.3% and Sp of 77.1%. Despite these encouraging results, the variance explained by the model is only 25% (Nagelkerke R2 = 0.254). Thus, although statistically significant, it cannot be validated, which is largely due to the small sample size. Nevertheless, based on these trends, the base-line values for CRP and ESR should be known prior to IVIG administration. The resistance predictor capacity highlights the role of inflammation in this disease, a possible underlying trigger in KD vasculitis.^21^

The etiology of KD remains uncertain, however, predisposing factors have been put forward. One, is the immaturity of the immune system, a theory that is supported by the fact that KD predominantly affects children under the age of five. In our study, 62.5% of the patients belonged to this age group, which, although corresponding to the majority of the sample, is below the 80% described in the literature.^1^ A possible explanation for this result is genetic contribution, since the incidences described in the literature are from studies with a wide range of ethnicities, including Asian children.

Coronary artery involvement occurred in 12 (25%) children, seven with ectasia and five with CAA. Therefore, the incidence of CAA was 10%, which is higher than the 4% reported in the literature. Comparing the groups with and without coronary artery involvement, a statistically significant difference was found regarding the duration of fever (p = 0.038). This result highlights the deleterious effects of persistent fever and the need for IVIG administration, preferably up to the tenth day of the disease, in order to avoid cardiac sequelae.^9^ The use of corticosteroids in KD is still a topic of debate and controversy. The most consensual is the use intravenous methylprednisolone (MPDN) at a dose of 15 to 30 mg/kg/day, for three days.^9^ In patients with refractory KD, MPDN supresses the inflammatory cytokine levels more quickly than a second dose of IVIG,^9^ although it is not recommended as a first-line treatment. Sleeper et al.^10^ evaluated the impact of corticosteroids at different times of the disease and showed that, with regards to the development of CAA, the only statistically significant difference was in those refractory to the first dose of IVIG which combined corticosteroids with the second dose of IVIG.

The cardiac complications and echocardiographic findings, others than coronary artery involvement were also evaluated. Three cases of left ventricular systolic dysfunction were identified, one of which with cardiogenic shock, a complication also described in the literature.^2^^,^^7^ In the acute phase, ten patients presented with pericardial effusion without hemodynamic compromise, three patients with mild mitral valve regurgitation and one with first degree AV block. Chbeir et al.^22^ found a relation between resistance to IVIG, CAA, and initial cardiac echocardiographic findings such as pericardial effusion, coronary hyperechogenicity, and coronary ectasia. The coronary hyperechogenicity and the lack of tapering on echocardiography were not considered as relevant factors, since they are subjective findings, poorly reproducible and can be found both in febrile illnesses and in healthy children.^23^ During the chronic phase, one patient remained with conduction system impairment and developed LV dilation, and another patient developed LV hypertrophy. The long-term cardiac repercussions in KD remain unclear. Friedman et al.^6^ reported an increase in the occurrence of long-term adverse cardiac effects, leading to primary angioplasty, coronary bypass surgery, heart transplantation, and death, in patients who developed CAA with higher z scores and who were initially resistant to IVIG. A study by Holve et al.^8^ revealed a low incidence of adverse cardiac effects in subjects up to 21 years of age, but a greater likelihood of developing high blood pressure from the age of 15.

Japanese predictive models presented poor clinical utility in this study (Table 4). The model with the highest specificity was Sano’s, although with very low sensitivity and with only a small number of cases included. Egami’s model was the most sensitive, however, not powerful enough to be validated. The genetic component may be the explanation for these differences. In fact, the results presented here are similar to those of other studies carried out outside Japan, in which none were able to validate the models in their samples.^10^^,^^14^^–^^17^^,^^20^ It is still important to note the differences in the study design in relation to the Japanese models, namely that they were applied only to patients with classical KD. Another Japanese study failed to validate the models in a sample exclusively composed of atypical KD cases.^24^ The Kobayashi model was validated for the Japanese population with Sn of 86% and Sp of 67%.^11^ Contrary to the present study, IVIG was administered at a dose of 1 g/kg on two consecutive days and resistance was considered if fever persisted 24 hours after the beginning of the treatment, or in case of recurrence after a period without fever. The Egami model was validated with Sn of 78% and Sp of 76%, however, resistance was considered if the CRP value did not decrease by more than 50% and fever persisted for longer than 48 hours after IVIG administration.^12^ Loomba et al.^25^ were not able to validate the Egami model even when applying it separately to classical and atypical KD, and by ethnicity. The Sano model, validated with Sn of 77% and Sp of 86%, was the only one of the three to adjust the size of the CAA to the body surface.^13^ However, it also used IVIG at a dose of 1 g/kg on two consecutive days and defined resistance if fever persisted 24 hours after the end of therapy.

The main limitations of this analysis are related to its retrospective methodology and sample size.

## Conclusions

CRP and ESR are independent variables that showed a predictive trend regarding resistance to IVIG, with optimal cut-off values of 15.1 mg/dL and 90.5 mm/h, respectively. However, there is a need for a multicenter study with a sample of adequate dimensions to validate a model based on these two analytical parameters. Cardiac complications are not limited to coronary arteries, and the study and follow-up of these patients should be more widespread. The validated models for the Japanese population have very limited utility in our population, further reinforcing the need and importance of new approaches.
